# Composite Fibrin and Carbon Microfibre Implant to Modulate Postraumatic Inflammation after Spinal Cord Injury

**DOI:** 10.3390/cells12060839

**Published:** 2023-03-08

**Authors:** Vincent Escarrat, Jimena Perez-Sanchez, Bilal El-Waly, Jorge E. Collazos-Castro, Franck Debarbieux

**Affiliations:** 1 Institut des Neurosciences de la Timone, Aix-Marseille Université and CNRS UMR7289, 13005 Marseille, France; 2 Centre Européen de Recherche en Imagerie Médicale, Aix-Marseille Université, 13005 Marseille, France; 3 Neural Repair and Biomaterials Laboratory, Hospital Nacional de Parapléjicos (SESCAM), 45071 Toledo, Spain; 4 Institut Universitaire de France, 75005 Paris, France

**Keywords:** two-photon microscopy, transgenic fluorescent mice, biocompatibility, neuroinflammation, spinal cord injury, natural materials

## Abstract

Poor functional recovery after spinal cord injury (SCI) drives the development of novel strategies to manage this devastating condition. We recently showed promising immunomodulatory and pro-regenerative actions of bio-functionalized carbon microfibres (MFs) implanted in a rodent model of SCI. In order to maximize tissue repair while easing MF implantation, we produced a composite implant based on the embedding of several MFs within a fibrin hydrogel. We used intravital imaging of fluorescent reporter mice at the early stages and spinal sections of the same animals 3 months later to characterize the neuroinflammatory response to the implant and its impact on axonal regeneration. Whereas fibrin alone was inert in the first week, its enzymatic degradation drove the chronic activation of microglial cells and axonal degeneration within 3 months. However, the presence of MFs inside the fibrin hydrogel slowed down fibrin degradation and boosted the early recruitment of immune cells. Noteworthy, there was an enhanced contribution of monocyte-derived dendritic cells (moDCs), preceding a faster transition toward an anti-inflammatory environment with increased axonal regeneration over 3 months. The inclusion of MF here ensured the long-term biocompatibility of fibrin hydrogels, which would otherwise preclude successful spinal cord regeneration.

## 1. Introduction

The management of sfigurepinal cord trauma requires taking control of the post-traumatic environment to limit inflammatory secondary damage, reduce neural cell death and tissue cavitation, and prevent the formation of dense connective tissue scars that would otherwise act as a physical barrier against spontaneous axonal regeneration [1]. Several strategies making use of biocompatible materials have been evaluated with the view of providing a substitutive trophic scaffold and metabolic supply for the regrowth of surviving severed axons [2,3].

Bio-functionalized conducting polymer-coated carbon microfibers (MFs) hold promise as biomaterials for developing advanced neuroprostheses and neural repair devices [4]. They can effectively serve as a multifunctional tissue scaffold, bridging spinal cord cavities and stimulating cell growth across the lesions. When modified with N-Cadherin or L1 neural cell adhesion molecules, direct neuronal attachment and axonal elongation are promoted on the MF surface [5]. When multimolecular complexes of poly-L-lysine, heparin, basic fibroblast growth factor, and fibronectin are attached instead, glial precursor cells migrate extensively on the MFs and indirectly facilitate axonal elongation [6]. The latter biofunctionalized MFs were implanted in mice and rats with SCI, providing evidence that they can enhance and guide the infiltration of the lesion by blood vessels, axons, and pro-healing immune cells [4,7].

In this regard, we have recently used intravital microscopy to investigate the complex immune-neural cell interplay during tissue repair after MF implantation [7]. Our results suggest that the biofunctionalized MFs inserted at the lesion site offer highways for faster immune cell infiltration besides aligned support for regrowing axons, and that the immune cell response may favor tissue regeneration. On the other hand, families of structured hydrogels have recently been injected at the lesion site to act as casting immunomodulatory materials [8] or as drug delivery systems [9]. Noteworthy, fibrin, a fibrous non-globular protein, is formed by the action of the protease thrombin on fibrinogen, which causes it to polymerize in a traumatic context. Fibrin has been used for the treatment of spinal cord injury due to its biological origin, mechanical properties, and capacity to accumulate and release various growth factors, therapeutic molecules, or even cells [10]. Fibrin is also considered an intrinsic, multifaceted regulator of inflammation, whose impact in a post-traumatic context critically depends on the engagement of selective binding partners within a specific microenvironment [11]. Enzymatic degradation of fibrin by plasmin finally releases fibrinogen degradation products (FDP) [12], which also exert context-dependent immunomodulatory roles [13]. The overall immunomodulation is all the more difficult to predict than fibrinolysis, which is dependent on the modulation of plasmin activity. Fibrinolysis is also dependent on the stabilization of fibrin mesh by the cross-linkage of its threads under the action of numerous factors, such as Factor XIII [11]. However, in a spinal cord injury context, experimental conditions have been recently established to obtain neurotrophic and guiding effects on regenerating axons with conformed fibrin hydrogels cast inside the lesion site [14,15].

The objective of the present study was to combine the two approaches in order to synergize their regenerative effect but also in order to ease the implantation of a large number of MFs at the lesion, thereby potentiating their trophic and immunomodulatory effects. As such, MFs were bundled inside a fibrin hydrogel matrix. The matrix allowed for the efficient handling and implanting of an organized MF assembly, which provided an additive immunomodulation to that of individual MFs. 

We performed long-term follow-up on the same triple fluorescent transgenic animals by intravital two-photon imaging as well as post-mortem histology at 3 months. Thanks to multicolor transgenic labeling of inflammatory cell populations, we separated the peripheral inflammatory component from the resident inflammatory response with the view to getting insight into the reported macrophage/microglia crosstalk responsible for the post-traumatic inflammatory response [16]. We have shown the spatio-temporal anti-inflammatory and pro-regenerative impacts of our composite implant compared to untreated conditions, but we have also outlined the evolving biocompatibility of the fibrin matrix according to the post-traumatic physiological status of the animal. Its excellent biocompatibility, observed at early stages, evolved into a deleterious activation of chronic inflammation, leading to impaired neuro-regeneration and even long-term neurodegeneration. Noteworthy, the inclusion of anti-inflammatory MFs inside the fibrin matrix allowed for benefit from its biomechanical properties while preventing the unreliable immunomodulatory effect of fibrin. Our results further emphasize the importance of animal models and long-term biocompatibility studies to reliably conclude on the suitability of any biomaterial to be incorporated into brain-machine prosthetic devices.

## 2. Materials and Methods

### 2.1. Animals

We operated 20 Thy1-CFP//LysM-EGFP//CD11c-EYFP triple heterozygous transgenic adult nine-weeks-old mice with multiple fluorescent cell populations [17]. These mice display a subpopulation of neurons with cytoplasmic expression of CFP (blue), neutrophils and monocytes with cytoplasmic expression of enhanced green fluorescent protein EGFP (green), as well as activated resident microglia and peripheral dendritic cells with cytoplasmic expression of enhanced yellow fluorescent protein EYFP (yellow). Mice were housed in cages with food and water ad libitum in a 12 h light/dark cycle at 22 ± 1 °C. Until the end of the protocols, food was supplemented with 4% agarose jelly containing 4% glucose. All experimental procedures were performed in accordance with the French legislation and in compliance with the European Community Council Directive of 24 November 1986 (86/609/EEC) for the care and use of laboratory animals. Our protocols on animals were authorized by the “Direction Départementale des Services Vétérinaires des Bouches-du-Rhône” (license D-13-055-21) and approved by the National Committee for Ethic in Animal Experimentation (Section N°14, Project APAFIS #4828-20 1504131125423 v2).

### 2.2. Preparation and Bio-Functionalization of Carbon MFs

The protocol for the preparation and bio-functionalization of carbon MFs was described previously [6,7,18,19]. In addition, poly(3,4-ethylenedioxythiophene)/poly-(styrenesulfonate)-comaleic acid) (PEDOT: PSS-co-MA) were electrodeposited on 7-µm-diameter carbon fibers (Goodfellow, Barcelona, Spain) by applying a constant anodic current of 1 µA/mm^2^ and a polymerization charge of 96 mC/cm^2^. Functionalization with bioactive molecules was performed on PEDOT: PSS-co-MA-coated carbon MFs (hereafter referred to as bare-MFs). Poly-L-lysine (PLL 57kDA; Sigma-Aldrich, Saint-Quentin-Fallavier, France) was bonded covalently to the carboxylic groups of the dopant. Heparin (10 mM; Sigma-Aldrich, Saint-Quentin-Fallavier, France) was dissolved in Phosphate Buffer Solution (PBS) and was then assembled on the PLL layer for 4 min. Subsequently, recombinant human basic fibroblast growth factor (bFGF, PeproTech London, UK, 100-18B) was applied at 1 µg/mL in PBS for 1 h. Finally, the MFs were incubated at 37 °C for 4 days with bovine fibronectin at 40 µg/mL (FN; Invitrogen, Barcelona, Spain, 33010-018) dissolved in PBS.

### 2.3. Preparation of the MF Bundle in a Fibrin/Thrombin Gel

In order to increase their stability and ease their implantation, MFs were bundled into a fibrin matrix. The bundles were prepared on the day of implantation. Teflon tubes were cut in half (1.2 mm segments) and placed into a petri dish to use as a template. Note that the scaffold was made under a binocular magnifier (M60, Leica-Microsystems, Nanterre, France). Fibrinogen (Sigma-Aldrich, Saint-Quentin-Fallavier, France, F1141) and thrombin (Sigma-Aldrich, Saint-Quentin-Fallavier, France, T7009) were previously solubilized in 10 mM HEPES buffer, and aliquots were kept at −20 °C until use, with respective final concentrations of 10 mg/mL for fibrinogen and 100 U/mL for thrombin. Fibrinogen and thrombin were warmed up to 37 °C and mixed in a ratio of 1:2. Upon mixing, they coagulated into a fibrin gel at the bottom of the Teflon template. Polymerization (or gelation) of fibrin was allowed for 5 min to obtain a solid gel before another layer was laid over it. Thereafter, 1.5 mm segments of functionalized MFs were placed on a layer-by-layer basis in between fibrin deposits.

Each layer included different steps in a specific order, as detailed for layer 1. First, 0.5 µL of thrombin was deposited (step 1), followed by the bio-functionalized carbon MF fiber (step 2), itself covered with 1μL of fibrinogen (step 3), and a 5-min wait for the fibrin polymerization (step 4) before starting the next layer. The number of MFs differed for each layer: 1 MF in the first layer, 2 MFs in the second, 3 MFs in the third, and 4 MFs in the fourth, for a total of 10 MFs per bundle. Care was taken to place the MFs parallel to each other with a minimal overlap. Note that similar layer-by-layer fibrin gels with no MFs were made to be used as controls. Finally, the rheological properties of fibrin, such as non-linear elasticity and soft compliance [20,21], make our implant modular but non-breakable, and thanks to its round shape and the non-adhesive properties of the Teflon hemi-tube, the fibrin bundle was detached by folding the edges and modeling with fine forceps.

### 2.4. Partial Unilateral Dorsal Quadrant Lesion (PUDQL) and Glass Window Implantation

The mice were anesthetized with intraperitoneal ketamine/xylazine (120 mg/kg; 12 mg/kg) and supplemented hourly with the same cocktail at a lower dose (40 mg/kg; 4 mg/kg). Following a dorsal midline incision over T12 to L2, the muscles between the spinal and transverse processes were resected using a scalpel. Animals were then suspended from a spinal-fork stereotaxic device (Harvard apparatus, Holliston, MA, USA). The dorsal musculature was further resected to expose the vertebrae, and the tips of modified staples were inserted along the edges of the T12 and L2 vertebrae and glued in place with cyanoacrylate. A modified paperclip was fixed to the staples to serve as a handle for surgery and imaging, and a layer of dental cement was applied to form a rigid ring to hold the vertebrae, staples, and paper clip together. A laminectomy was performed by removing two spinal processes from the exposed vertebrae. A partial unilateral dorsal quadrant lesion (PUDQL) was made on the rostral edge of the exposed spinal cord. To do so, a 26 G needle blade was used to make a 1-mm longitudinal incision on the right side of the spinal cord, as close as possible to the central vein and the spinal cord and as deep as the shaft of the needle. The spinal cord was then transected using a pair of fine-angled Vannas spring scissors (Fine Science Tools, Heidelberg, Germany) from the rostral end of the incision to 0.5 mm lateral and approximately 0.4 mm deep from the dorsal surface. A second, identical incision was made from the caudal end of the initial incision. A final horizontal cut was made from the injured side toward the dorsal vein, and the tissue between the rostral and caudal transections was removed. The lesion was made so that the caudal incision reached the middle of the window and the rostral incision was 200 microns away from the most frontal edge of the window. Special care was taken to not cut the central vein so that bleeding was kept minimal. MFs bundles or fibrin/thrombin gels only were then implanted into the lesion, in a caudal-to-rostral motion.

A line of liquid Kwik-Sil (World Precision Instruments, France) was applied along the midline of the spinal cord, and the glass window was immediately glued and cemented over the spinal cord. Kwik-Sil was used to seal the lesion site to the glass window to prevent fibroblastic invasion. Kwik-Sil was applied on the « dried » lesion site using the mixing application tip that ensures chemical reaction between the two components of the tube, hence hardening of the silicone. The glass window had to be perfectly clean to allow adhesion between the silicone and the glass. Post-operative analgesia was obtained by the administration of cortamethasone (0.2 mg/kg) and rimadyl (5 mg/kg) immediately following surgery and every two days for 10 days after surgery. The mice did not require manual bladder emptying since the lesion affected only the most dorsal tracts. The animals were split into 4 groups: animals without the lesion were named WO (Window Only), PUDQL was only named LO (Lesion Only), and PUDQL and the fibrin were only named FO (Fibrin Only), PUDQL and the fibrin+MFs bundle were named BMFs (Bundle of MicroFibres).

### 2.5. Intravital Imaging

The same mice were imaged three days (D3) after window implantation to target the peak of peripheral cell infiltration. For each imaging session, mice were lightly anesthetized with 1.5% isoflurane (*v*/*v*) in the air for 2 min, followed by intraperitoneal ketamine/xylazine (100 mg/kg or 10 mg/kg) administration. The animals were supplemented with 0.4–1.0% isoflurane (*v*/*v*) in the air for 45 min after the start of the session until completion. Throughout imaging, the animals were freely breathing, and the microscope chamber was warmed to 32 °C to maintain the body temperature at 37 °C. Following each imaging session, the animals were returned to their cage with a piece of tissue for nesting and kept warm until they recovered from anaesthesia. A tuneable femtosecond pulsed laser (Ultra II Chameleon Coherent, Lisses, France) was coupled to a Zeiss two-photon (2P) microscope (LSM 780, Carl-Zeiss France SAS, Rueil-Malmaison, France) equipped with a 20× water immersion objective lens (NA = 1.0) and five non-descanned detectors. The laser was tuned to 940 nm to optimize the simultaneous excitation of the labeling fluorophore combination while minimizing the heat accumulation by implanted carbon MFs. Filter sets were designed to optimize the separation of the emission spectra of multiple fluorophores. For each image stack, laser intensity was adjusted according to imaging depth in order to maximize signal intensity while minimizing saturation throughout the image stack. A second harmonic signal reflected by superficial collagen fibers was used to identify meninges. Blood vessels and remarkable axon patterns were used as anatomical markers to find the region of interest for each animal. Tiled-stack images were acquired with a field of view of 424 × 424 µm and an optical sectioning of 3 µm over a depth of ~80 to 100 µm below the meninges. Micro adjustments during mouse positioning allowed the imaging of the same volume of interest throughout imaging sessions. The volume, mainly lying between 20 and 50 µm was used for quantitative analysis.

### 2.6. Histological Study

Ninety days after PUDQL (D90) mice were perfused with 4% paraformaldehyde. Then, spinal cords were removed, post-fixed overnight in 4% paraformaldehyde, and cut into 50 µm thickness coronal sections and 100 µm thick sagittal sections using a vibratome (Leica Microsystem, Rueil-Malmaison, France). Spinal cord slices were then mounted on glass slides with coverslip. Imaging was performed on a Carl Zeiss LSM780 confocal microscope using the 458 nm and 514 nm wavelengths to excite the CFP-Thy1^+^ and EYFP-CD11c^+^ signals; detection was obtained using 469–516 and 517–544 nm band pass filters, respectively. Tiled-stack images were acquired with an optical sectioning of 5 µm over a depth of 25 µm. Late-stage inflammation, referred to as chronic inflammation [7], was thus evaluated from a set of five coronal 50 µm thick slices centered on the lesion epicenter and spreading rostro-caudally over 2.4 mm at 600 µm intervals in 2 animals per condition.

### 2.7. Image Analysis

Images were analyzed using ZEN 2.1 (Zeiss, Jena, Germany), ImageJ software, and Arivis Vision 4D software (Arivis AG, Berlin, Germany). ZEN 2.1 was used for post-acquisition spectral unmixing, and analysis was performed on the resulting unmixed data. 

The intravital images were analyzed using the protocol described in [7]. The presented images are pseudo-colored and contrast-enhanced for clarity. 

On fixed spinal cord sections, quantification was performed only on the EYFP channel of confocal Z-stack fluorescent images. An automated 3D segmentation pipeline was designed using the Arivis machine learning toolbox. Immune cells were counted in the total 3D volume of the confocal acquisitions using the Arivis software’s machine learning object segmentation, which allowed the automatic detection of the two populations of CD11c^+^ cells in a few minutes in the whole volume. This method required a preliminary machine learning training on ten mixed images where more than 200 cells of each type were drawn by hand to adjust feature settings (intensity, edge texture, orientation). An algorithm was trained to distinguish two subtypes of microglial CD11c^+^ cells based on their round or ramified morphologies and their densities quantified. Following cell segmentation, the 3D spinal cord images were regionalized into six equal regions evenly distributed relative to the central canal. Grey and white matter were also outlined both on the ipsi- and contra-lateral sides. The pipeline thus provided a detailed description of the 3D distribution of each microglial subpopulation. Quantifications presented in this study express the average density of cells evaluated per volume unit (of approximately 0.01 mm^3^).

### 2.8. Statistics

All data are expressed as mean ± SEM. Statistical analysis was performed using GraphPad Prism software for Mann–Whitney tests, and * *p* < 0.05, ** *p* < 0.01, *** *p* < 0.001, **** *p* < 0.0001 were considered to have statistical significance.

## 3. Results

### 3.1. Tailoring a Fibrin/Carbon Based Implant

**Figure 1:**
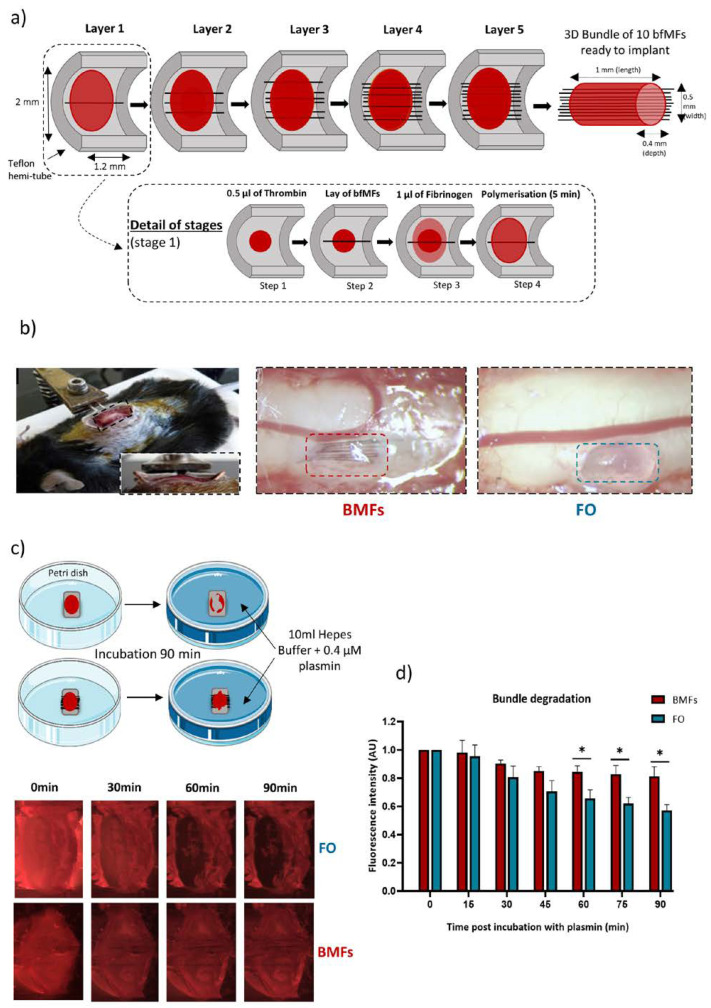
Preparation and characterization of the neuroprosthetic implant (**a**) Embedment of MFs in fibrin gel. The composite implant was obtained by successive layers of small volumes of fibrinogen-thrombin mixture (1:2) interspaced by biofunctionalized MFs. A teflon hemi-tube was used as support for the fabrication. (**b**) For intravital experiments, the fibrin-based implants were positioned inside the lesion cavity immediately after injury. Bright field images were taken prior to glass window placement for BMFs or FO implants. (**c**) To evaluate fibrin degradation with or without bfMFs, the implant was immersed in a bath containing 0.4 μM of plasmin diluted in HEPES buffer. The kinetics of plasmin induced fibrinolysis was assessed by incorporating fluorescent sulforhodamine B in the fibrinogen/thrombin mixture and measuring the fluorescence of the formed gel. Fluorescence images were compared every 30 min in FO (top) and BMFs conditions (bottom). (**d**) Evolution of the normalized fluorescence intensity at the center of the implant. Graph represents the mean of n = 3 similar experiments. * *p* < 0.05.

The fibrin-based implant consisted of a succession of layers of fibrinogen/thrombin, and MFs in order to obtain a 3D bundle that conformed as much as possible to the shape of the PUDQL (Figure 1a). Based on the protocol detailed in the method section, we obtained a final implant with approximately 1 mm of length, 0.5 mm of width, and 0.4 mm of depth, matching the size of the lesion. These implants, either comprising 10 bio-functionalized MFs (Bundle of MFs, BMFs) or without MFs (Fibrin Only, FO), were introduced freshly prepared into the spinal cord immediately after injury, as shown in Figure 1b.

### 3.2. Plasmin Mediated Resorption of the Implant 

Prior to their intravital implantation, an in-vitro study was conducted to evaluate the robustness of such implants to enzymatic degradation by plasmin at room temperature and at 37 °C to mimic intravital conditions. FO and BMFs implants were incubated for 90 min in a bath containing 0.4 μM of plasmin (Sigma; P1867; 3.9 units/mg) diluted in HEPES buffer (Figure 1c). Sulforhodamine indicator (1 mg/mL) was added to the thrombin and fibrinogen solutions prior to mixing. To evaluate the degradation, trapped fluorescence intensity inside the red bundle was monitored every 5 min for 90 min as an index of fibrin digestion. Images taken at regular intervals following the immersion into the plasmin bath (Figure 1c) showed that the FO were degraded over time while the BMFs seemed comparatively stable. The quantification of fibrin degradation by plasmin in both FO and BMF conditions is presented in Figure 1d. We found that more than 40% of the total fibrin was digested within 90 min incubation in plasmin. However, inclusion of a MF scaffold inside the fibrin implant reduced its sensitivity to plasmin digestion by a factor of 2, with fluorescence declining only by 20% over 90 min. These data suggested that the MFs would slow down the resorption of the fibrin gel in vivo. 

### 3.3. Immunomodulatory Effects of the Composite Implant after Spinal Cord Injury

**Figure 2:**
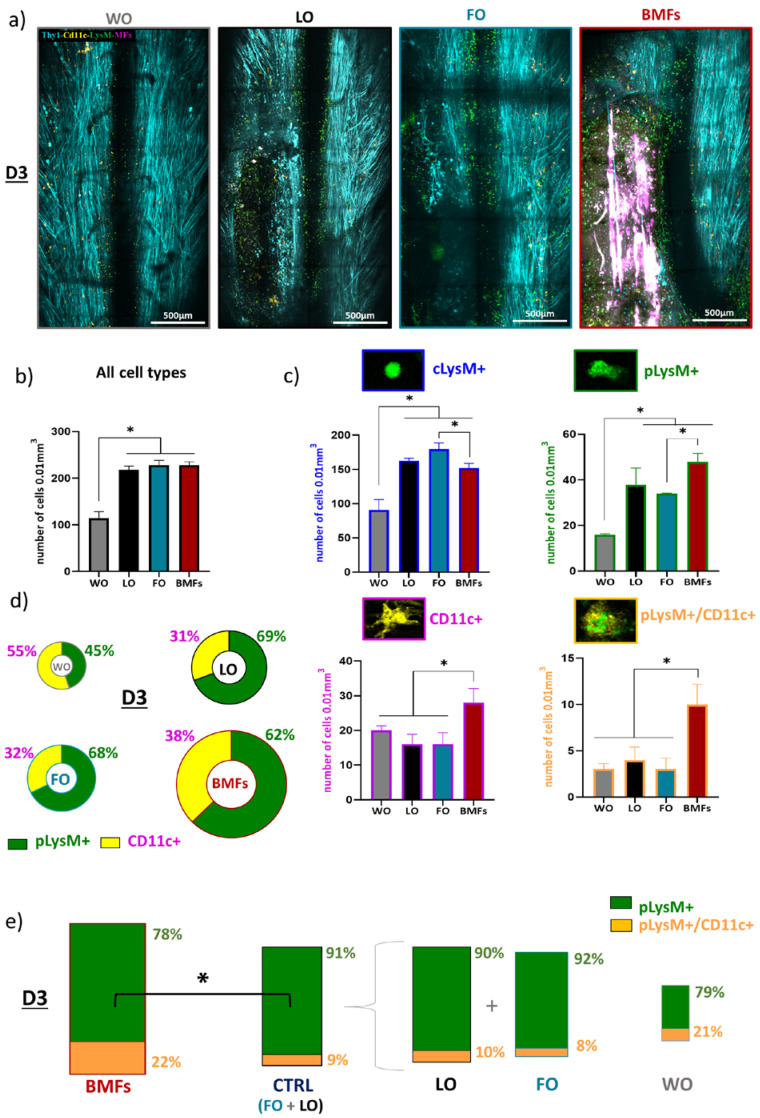
Characterization of immune cell responses in BMFs, FO, LO, WO triple fluorescent mice at three days after surgery. (**a**) Representative maximal intensity projection of 2P z-stack images obtained in vivo three days (D3) after surgery in Thy1-CFP//LysM-EGFP//CD11c-EYFP triple transgenic mice under various conditions; lesion treated with bundle of MFs (BMFs), lesion treated with fibrin matrix only (FO),—lesion only (LO) and—non-injured control mice implanted with the glass window only (WO). (**b**) Quantitative representation of global immune cell densities (number of cells/0.01mm^3^) in each condition on D3. (**c**) Quantitative representation of the densities of immune cell subtypes: cLysM^+^, pLysM^+^, CD11c^+^ and pLysM^+^/CD11c^+^ double-labeled cells. (**d**) Circular graphs showing the relative percentage of infiltrated pLysM^+^ versus CD11c^+^ cells at D3 after surgery in each condition. Diameters of the circles are proportional to the total number of pLysM^+^ and CD11c^+^ cells densities. Note the significant increase of inflammation in the presence of MFs. (**e**) Bar graphs showing the relative percentage of pLysM^+^/CD11c^+^ double-labeled cells among total pLysM^+^ cells at D3. Areas of the boxes are proportional to the pLysM^+^ cell densities. Note that in addition to their numbers, the proportion of pLysM^+^/CD11c^+^ double-labeled cells was significantly increased in BMFs compared to FO and LO. For each data, results are displayed as mean ± SEM, n = 4 animals, * *p* < 0.05.

In order to assess the role of fibrin-based implants on the dynamics of early post-traumatic inflammatory processes occurring early after PUDQL, we first quantified immune cell numbers and densities on 2P intravital images obtained at three days (D3) after surgery in BMFs, FO, LO, and WO Thy1-CFP//LysM-EGFP//CD11c-EYFP triple transgenic mice (Figure 2a).

Four different cell populations were identified from LysM-EGFP and CD11c-EYFP labeled cells, as previously detailed in [7]: (1) Circulating cells (cLysM ^+^, EGFP) were found inside and in the vicinity of blood vessels and corresponded to circulating monocytes and neutrophils; (2) once infiltrated inside the spinal cord tissue, these cells underwent morphological changes and were referred to as EGFP expressing parenchymal LysM^+^ cells (pLysM^+^), composed mainly of granulocytes and monocyte-derived cell subtypes; (3) among these, double-labeled (pLysM^+^/ CD11c^+^) monocyte-derived dendritic cells (moDCs), expressing both EGFP and EYFP, they represented a specific subset of peripheral cells that had differentiated in situ from the infiltrated pLysM^+^ monocytes [17,22]; (4) pure EYFP expressing CD11c^+^ cells represented microglial cells in their activated state. Peripheral and resident components of the neuroinflammatory response could thus be examined.

Global inflammation was characterized by the automatic computational segmentation of these fluorescent cell populations, as previously reported [7]. Quantitative analysis of cell density showed that PUDQL systematically induced a doubling of the global inflammatory response (including parenchymal and circulating cells) compared to window implantation alone (WO), irrespective of the treatment condition (Figure 2b). The early response to PUDQL was characterized by the recruitment of peripheral LysM+ cells as well as an increase in resident CD11c+ microglial cells, but two-thirds of the intraparenchymal inflammatory cells were in fact of peripheral origin (Figure 2c,d). Importantly, at this stage, the implant made of only fibrin (FO) had no impact on the post-traumatic inflammation, both in terms of the intensity of the response and the contribution of the different inflammatory cell populations when compared to the lesion alone (LO) (Figure 2c). 

By contrast, inclusion of MFs in the fibrin gel (BMFs) boosted both the microglial accumulation and the infiltration of peripheral immune cells inside the lesion (Figure 2c). CD11C+ cell density increased by 100% (BMF: 28 ± 4 cells/vol versus FO: 16 ± 3 cells/vol; Figure 2c), while the density of infiltrated pLysM^+^ monocytes and neutrophiles (48 ± 3 cells/vol against 34 ± 1 cells/vol) increased by 50%. Noteworthy, this increased infiltration of pLysM^+^ was coincident with a significant reduction of their circulating densities (BMFs: 152 ± 7 cells/vol versus FO: 180 ± 9 cells/vol) (Figure 2c,d), suggesting that MFs likely acted as a breach inside the bulky fibrin gel, offering highways for parenchymal invasion by peripheral immune cells. 

However, the most striking effect of the presence of MFs at this post-traumatic stage was the increase by a factor of 3 in the densities of pLysM^+^/CD11c^+^ moDC cells (BMFs:10 ± 2 cells/vol versus FO: 3 ± 1 cells/vol, Figure 2c), which accounted for 22 ± 4% of all infiltrated pLysM^+^ cells in BMFs animals instead of 9 ± 3% of pLysM^+^ cells in control animals (FO and LO) (Figure 2e).

Altogether, our results thus showed that implantation of fibrin alone into the lesion site did not influence the inflammatory response at an early stage after a PUDQL lesion, while implantation of MFs promoted early activation of microglia and also increased recruitment of monocytes, whose differentiation into moDCs was boosted. 

### 3.4. Fibrin Degradation Precludes Longitudinal Intravital Imaging

**Figure 3:**
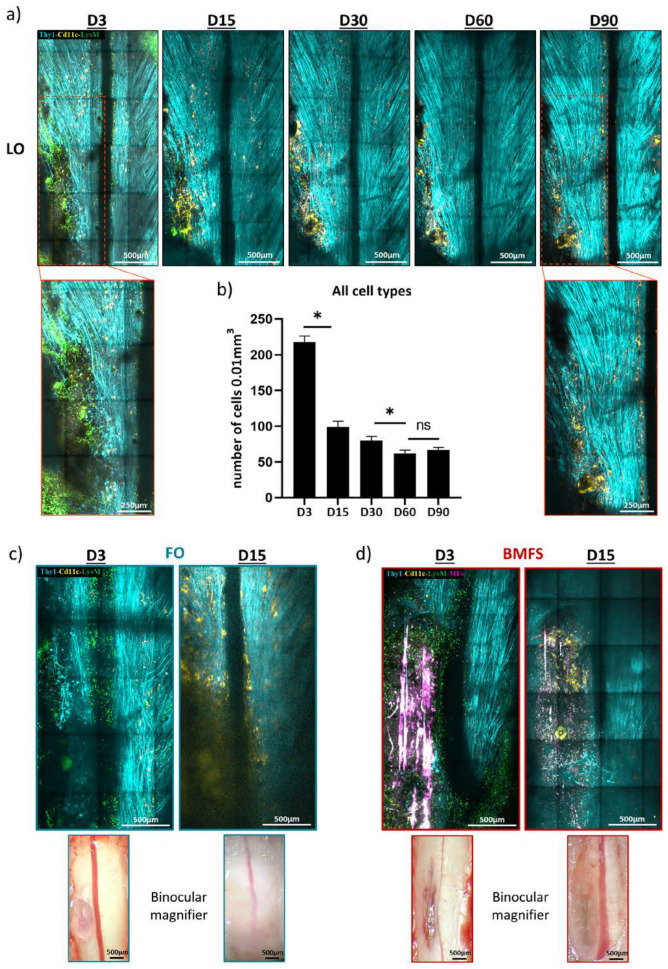
Degradation of the fibrin matrix triggers fast opacification of the window precluding longitudinal intravital 2P imaging. (**a**) Representative maximal intensity projection of 2P z-stack images obtained in vivo on the same animal, three (D3), fifteen (D15), thirty (D30), sixty (D60) and ninety (D90) days after injury in LO animals. Note that the glass window remains clear over time. Comparison of zoomed (red squares) images at D3 and D90 showed the axonal regeneration (**b**) Global immune cells density for LO mice at D3, D15, D30, D60, D90 after surgery. (**c**,**d**) Representative maximal intensity projection obtained in vivo on the same animal, at D3 and D15 for FO (**c**) and BMFs (**d**) mice with their corresponding binocular magnifier bright field images. Note the opacity of the glass window caused by fibrin degradation and associated cell responses. Both bright field and 2P images show that turbidity is far more pronounced for FO than for BMFs animals at D15. (n = 3, * *p* < 0.05).

Implantation of a dorsal glass window immediately after a spinal cord lesion allowed for repeated 2P imaging sessions with optimal microscopic resolution over several months in LO animals (Figure 3a).

The quantification of inflammatory cell densities at two-week intervals confirmed the spontaneous recovery of the inflamed tissue within two months (Figure 3a,b). Indeed, we observed that the density of cells decreased significantly and continuously from D3 (218 ± 8 cells/vol) to D60 (62 ± 5 cells/vol). Noteworthy, there were no cell density variations occurring between D60 (62 ± 5 cells/vol) and D90 (67 ± 4 cells/vol), suggesting that the spinal cord cellular microenvironment remained stable from the second month post-injury. However, the magnified images of the lesion site at D3 and D90 (red squares in Figure 3) illustrate qualitatively the kinetics of inflammation, with the initial peripheral pLysM^+^ inflammation giving progressive place to a chronic CD11c^+^ cell accumulation along with recovery of axonal densities. 

Additionally, in the case of FO and BMF implanted animals, the initial transparency of the fibrin implant evolved toward turbidity as a sign of gel degradation and infiltration by fibrotic tissue (Figure 3c,d). This phenomenon precluded subsequent resolutive cellular imaging from the second week onward. Therefore, neither immune cell phenotypes nor axonal regeneration could be studied in vivo after 2 weeks in animals with fibrin-based implants. However, image degradation was more obvious in FO animals (Figure 3c,d), supporting the idea of accelerated fibrinolysis as demonstrated in vitro. Consequently, the long-term impact of these implants was studied post-mortem on fixed spinal cord slices after a 90-day post-traumatic healing period.

### 3.5. Chronic Inflammatory Responses to the Different Types of Implants

**Figure 4:**
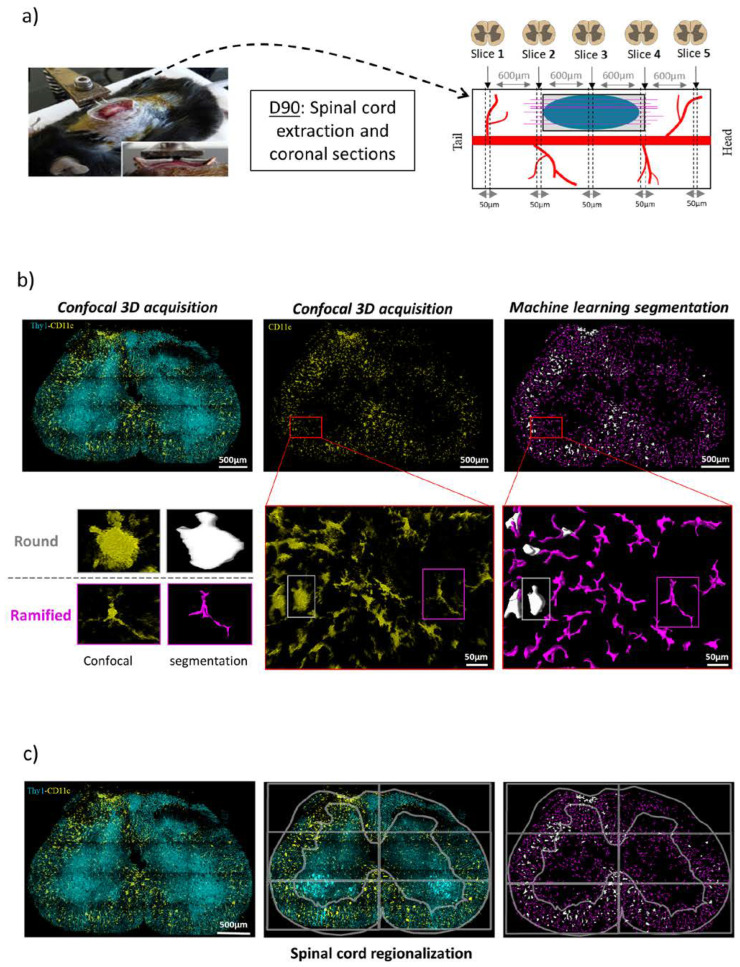
Automated machine learning segmentation protocol reveals two morphologically distinct populations of CD11^+^ cells in spinal cord sections on D90. (**a**) On D90 the chronic inflammatory status was evaluated from coronal 50 µm thick spinal sections taken every 600 µm on both side of the lesion epicenter. Scheme represents the spinal cord with its blood vessels (red), lesion area (grey), MFs (pink) embedded in a fibrin bundle (blue). (**b**) Representative confocal images showing Thy1-CFP neurons and CD11c^+^ cells (left), or CD11c^+^ cells only (middle); two classes (white and purple) segmentation (right) of the raw image (middle) obtained after 3D machine learning pipeline on Arivis software. Morphology of round miroglia (white) and ramified microglia (purple) is illustrated in zoomed insets from the outlined regions of interest (red). (**c**) Round (white) and ramified (purple) CD11c+ cells were systematically identified in the slice and their densities quantified in remarkable zones of spinal cord. Six squared regions of interest (ROI) were overlaid to define dorsal, medial and ventral regions in ipsi or contralateral location of the lesion. For each ROI, the location of cells in the white or grey matter was further evaluated.

At 3 months post-trauma, only CD11c^+^ microglial cells were present, but two subtypes of microglial CD11c^+^ cells could be distinguished based on their round or ramified morphologies (Figure 4b).

**Figure 5:**
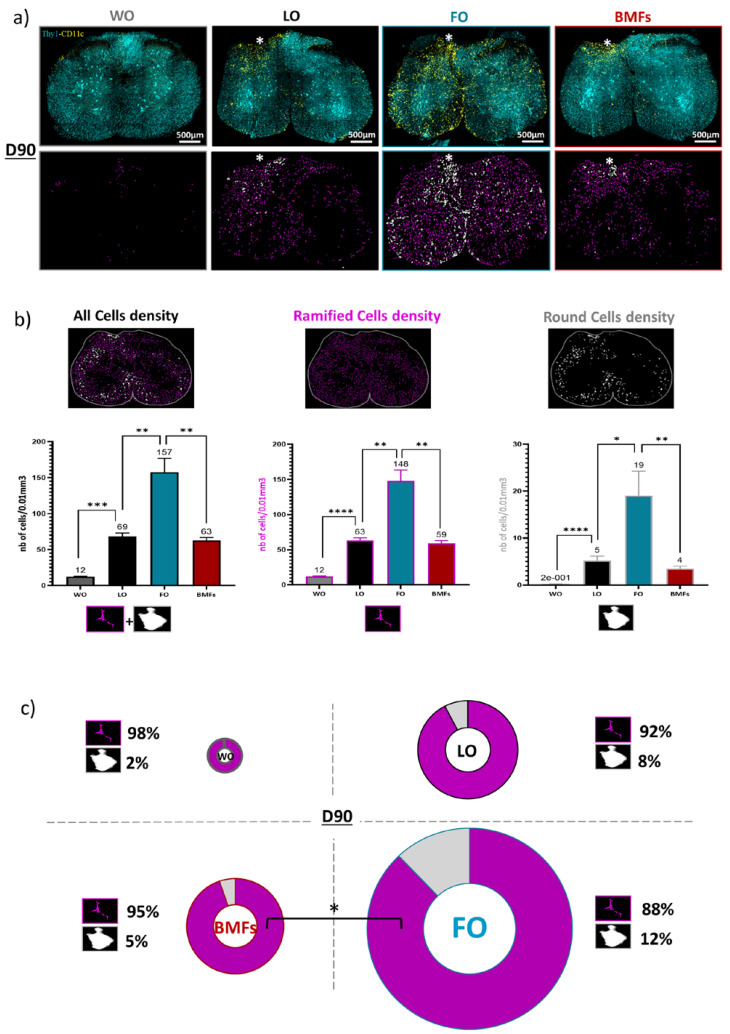
Quantification of chronic inflammation ninety days after lesion (**a**) Representative confocal (top) and related segmented (bottom) images for coronal spinal cord section obtained on D90 from BMFs, FO, LO, WO triple-transgenic mice. (**b**) Bar graphs of CD11c^+^ cell densities (number of cells/0.01 mm^3^) depending on their morphology (**c**) Circular graphs showing the relative proportion of Round versus Ramified cells on D90 in each condition. Diameters of the circles are proportional to the total CD11c^+^ cells densities. For each data, results are displayed as mean ± SEM, n = 10 (5 slices from 2 animals), * *p* < 0.05, ** *p* < 0.01, *** *p* < 0.001, **** *p* < 0.001.

CD11c^+^ microglia were more numerous in FO animals compared to all other conditions on D90 (Figure 5a). Quantification showed that their density was more than threefold higher in the spinal cord of FO animals than in the LO animals (Figure 5b), although fibrin was initially inert according to the inflammation levels observed on D3 (Figure 2). Both round and ramified subtypes of microglia were enhanced in the presence of only fibrin (Figure 5a,b). However, round microglia were significantly more numerous, and their density increased by fourfold (FO: 19 ± 5 cells/vol; versus LO: 5 ± 1 cells/vol) to reach approximately 12% of the whole CD11c^+^ population (Figure 5b,c). Such microglial cells were absent from the resting state spinal cord of uninjured WO animals (Figure 5a–c).

Furthermore, the inflammatory environment of the BMF animals was similar to that of the LO animals (Figure 5a–c). MFs were then able to counteract the chronic inflammation normally observed in FO animals. In addition to the quantity of cells, observations (Figure 5a) clearly revealed a distinct spatial distribution of CD11c^+^ cells relative to the lesion site between the LO, BMFs, and FO mice. Then, based on the machine learning segmentation (Figure 4b,c), we precisely assessed the round and ramified CD11c^+^ cell distributions in the spinal cord. 

**Figure 6:**
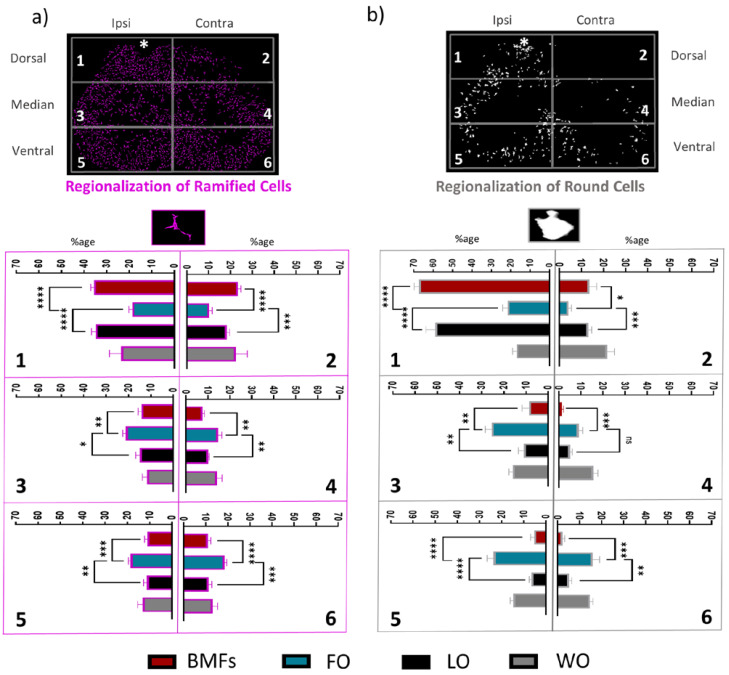
Dorsal and ipsilateral regionalization of chronic inflammation on D90 post lesion. (**a**) For each of the six rectangle ROIs, percentage of the total number of ramified CD11^+^ cells present in the section in each condition. (**b**) Same as (**a**) for round CD11^+^ cells. The white star indicates the lesion site. Results are displayed as mean ± SEM, n = 10 and * *p* < 0.05, ** *p* < 0.01, *** *p* < 0.001, **** *p* < 0.001.

The chronic inflammation reported in the FO mice was spatially extended to all the spinal cord since the distribution of both ramified and round cells was uniform, with approximately 15% of cells in each of the 6 defined ROIs (Figure 6a,b), including areas contralateral to the lesion. By contrast, the very significant lower number of both round and ramified microglia in BMFs animals was accompanied by the clustering of most microglial cells in ROI 1 (lesion area). The phenomena were particularly pronounced for the round cell subpopulation, of which 68 ± 4% of cells localized in ROI 1 for the BMFs mice *versus* only 21 ± 3% for the FO mice (Figure 6a,b). Altogether, these observations suggested a spread of inflammatory signals issued from the fibrin that MFs were able to counteract.

### 3.6. The Composit Fibrin-MF Implant Improves the Axonal Regeneration

**Figure 7:**
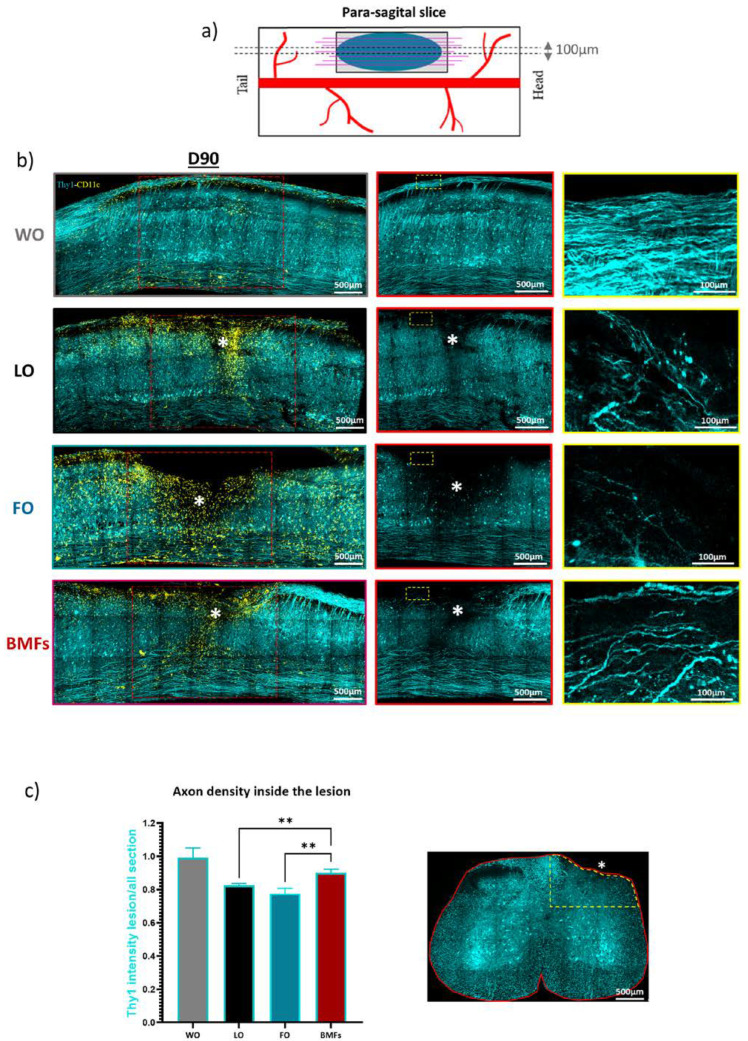
Rostro-caudal distribution of chronic inflammation and axonal densities on D90 post lesion. (**a**) The rostro-caudal distribution of chronic inflammatory status was evaluated from parasagittal 100 µm thick spinal sections taken in the center of lesion as shown in scheme. (**b**) Representative Maximum Intensity projections of multicolor confocal z-stack images from para sagittal spinal cord slices in each condition. Axonal networks (Thy1-CFP) inside the red square ROIs are presented in the middle panel; zoomed view of the yellow rectangle ROIs are shown on the right panel. White star indicates the lesion site. Note the spreading of CD11^+^ cells along the rostro-caudal axis as well as the orientation of axonal regrowth for each condition (BMF; FO; LO; WO). (**c**) Ratio of the average CFP fluorescence signal in the ipsilateral dorsal ROI against the average fluorescence in the corresponding contralateral ROI was used as an index of axonal regeneration. This index is close to 1 for WO animals where no axonal degeneration is expected. The white star represents the lesion site. For each data in this figure, results are displayed as mean ± SEM, n= 10 (5 slices, 2 animals). ** *p* < 0.01.

In order to evaluate the neuronal correlates of the immunomodulation induced by the implants, the spinal cord of one animal per group was sliced parasagittally (Figure 7a) to better visualize the axons orientation. Thy1^+^ blue axons and CD11c^+^ yellow microglia were imaged by dual color confocal microscopy. 

Hyperactivation of microglia was confirmed in the rostro-caudal sections of the FO animal after 90 days (Figure 7b). CD11c^+^ cells were in this case evenly distributed along both the rostro-caudal and the dorso-ventral axes, contrary to the focal accumulation observed in the dorsal part of the lesion epicenter in LO animals (Figure 7b). The microglial distribution pattern in BMF animals was similar to that in LO animals, supporting the anti-inflammatory action of the MFs in counteracting the fibrin induced microglial activation. 

At the level of axons, a larger and deeper lesion site was visible at D90 in the FO animal compared to the milder lesions observed in both LO and BMFs animals. Higher magnification images further highlighted the sparseness of axons inside the FO lesion instead of the numerous though tortuous axons seen at D90 in the LO lesion (Figure 7b zoom). In BMF’s lesion, axonal density was highest despite the presence of fibrin; axons furthermore appeared straighter and more longitudinally oriented, contrary to the LO condition. 

The observations made on the sagittal slices were finally confirmed on coronal slices from 2 extra animals per group. Average axonal densities inside the lesions were indeed evaluated in both coronal and sagittal slices for each condition (Figure 7c). Whereas severed axons spontaneously regenerated in all the injured animals, our data indicated that casting the lesion cavity with fibrin on the day of trauma was responsible for the long-term impediment of spontaneous recovery. Embedment of MFs inside the fibrin matrix was sufficient to counterbalance the detrimental effect of fibrin alone and to benefit from the anti-inflammatory, pro-regenerative, and guiding effects of MFs on axonal regeneration compared to untreated LO conditions.

## 4. Discussion

### 4.1. Implantation Strategy

In this study, we implanted a bundle of 10 biofunctionalized carbon MFs embedded in a fibrin hydrogel matrix as a proof of concept for an easy-to-implant tailored scaffold to repair SCI in mice. By scaling up the number of implanted MFs from our original protocol [7], we aimed at maximizing the MF induced immunomodulation that we believed was responsible for the accelerated resorption of chronic inflammation and improved regeneration of axonal networks. However, because the serial implantation of individual fibers inside the lesion site was tedious, time inefficient, and also prone to MF breakage upon misguided surgical gestures, we have worked out a high-throughput implantation protocol that is suitable for the preclinical management of SCI and that is scalable for large subjects.

### 4.2. Interest of Biofunctionalized Carbon MFs

The conducting polymer-coated carbon MFs used in this study have the potential to be part of reparative neuroprosthetic devices because of their ability to deliver or record electrical currents in the CNS parenchyma [18,19]. Their small diameter and reduced stiffness compared to conventional metal or silicone electrodes ensure minimal tissue damage upon implantation [23]. These MFs were biofunctionalized with several protein layers to promote axonal growth and glial cell migration in vitro [5,6] and were subsequently used to improve neuroglial interactions and axonal regeneration after SCI in rats and mice [6,7]. Optimal biocompatibility was observed over several months, and MFs promoted axonal regrowth in their vicinity. Additionally, the MFs initially promoted inflammatory cell recruitment, possibly through a fibronectin-dependent mechanism. In a second stage, they induced a quick differentiation of infiltrated monocytes toward moDC with a polarization of macrophages and microglia in a M2 pro-healing phenotype [7], likely through bFGF signaling [24]. Our present data confirm those preliminary observations and show that increasing the number of MFs significantly increases their impact on inflammation at both early and later stages after implantation. These dose-dependence effects are likely attributable to the fibronectin and bFGF coating of the MFs. Therefore, and as expected, increasing the number of MFs allows to ameliorate the inflammatory post-traumatic environment and consequently the tissue repair.

### 4.3. Fibrin, an Ideal Matrix? 

Embedment of a bundle of MFs into a solid matrix thus allowed implantation of regularly interspaced MFs as well as their mechanical protection during surgical handling. Although alginate had initially been tested as a matrix for MF implantation [4], it was abandoned due to its calcium binding properties and the subsequent dysregulation of the traumatic environment. Although innovative synthetic organic materials might have been considered substitutes [8], we rather looked for biological materials with granted biocompatibility and the ability to play a role in repair strategies by casting traumatic cavities. Fibrin mesh contributes to blood clotting and wound healing and has already been used as an exogenously applied casting material in post-traumatic cavities [10]. The mechanical properties of the fibrin gel can be adjusted to match those of the surrounding spinal tissue and to minimize secondary mechanical damages. Although tissue compression and shearing were not a critical issue in our conditions where spinal cord movements are cancelled by the cemented glass window, the fibrinogen/thrombin ratio can be eventually adjusted to fit the expected clinical application [12,14]. Fibrin hydrogel is endogenously resorbed by enzymatic degradation over a few weeks, expectedly promoting the progressive reconstitution of the physiological environment [25,26] and offering possibilities to release pharmacological compounds or signaling molecules initially soaked inside the gel [9]. 

In the present study, we verified in vitro the enzymatic degradation of fibrin by plasmin.

These experiments clearly revealed that the MFs slow down fibrin degradation kinetics. The faster opacification of the glass window in FO animals further confirmed this observation in in vivo conditions. Whereas blood flow shear forces have been shown to induce laminar polymerization of fibrin threads rather than random polymerization [27], we propose that the presence of MFs during fibrinogen and thrombin deposition likely enforced changes in fibrin structural properties (diameter, orientation, etc.) and therefore changes in fibrinolysis kinetics [12,28,29]. In addition, the better stability of the fibrin gel in the presence of MFs may result from the interaction of fibrin with fibronectin [30], the cell adhesive protein used in the last coated layer of the MFs bio-functionalization.

Fibrinogen is known to induce microglial activation responsible for neurodegeneration, neuroinflammation, and autoimmunity in animal models of CNS disease [31,32]. Fibrin assemblies themselves and their degradation by plasmin result in the release of fibrinogen degradation products (FDP) that have context-dependent immunomodulatory roles [12,13]. Indeed, fragments D and E, the degradation products of fibrin and fibrinogen by plasmin, can have pro-healing activity [12], but they also attract neutrophils [33] and promote the extravasation of circulating monocytes through endothelial junctions [13]. Fibrinolysis induced immunomodulation is thus a highly dynamic phenomenon whose outcome largely depends on the pathophysiological context. 

Our experimental protocol allowed us to evaluate over time the effects of fibrin and fibrinolysis in our FO and BMF animals where fibrin had been exogenously introduced. In FO animals, fibrinolysis induced chronic inflammation and was deleterious for axons; in BMF animals, where fibrin is more resistant to fibrinolysis, these deleterious effects were prevented. In that respect, it seems that in the context of a spinal cord lesion, the pro-inflammatory FDP impact depends on the kinetics of their release and the concentration of FDP at different stages of the post-traumatic neuroinflammatory cascade.

Thus, fibrin could be an ideal matrix as support for in vivo neuroprosthetic implants only if its degradation rate is carefully controlled to match that of pro-healing cellular responses. Consequently, fibrin gel structure appears to be a key parameter to regulate immunomodulation. In our study, the biofunctionalized carbon MFs gave structure to the fibrin and provided both mechanical support and chemical stimuli to foster tissue repair, likely counteracting the negative effects of FDP and even facilitating their positive effects on the restoration of the spinal cord.

### 4.4. Imaging to Study Biocompatibility and Cell Responses to Implants in the CNS

Our longitudinal in vivo cellular imaging study carried out inside the spinal cord was technically more difficult than those usually conducted in the cortex to evaluate biomaterials. Indeed, external myelin coverage makes the spinal cord more sensitive to inflammation and prevents deep imaging compared to the cortex. Although we previously successfully achieved longitudinal imaging throughout 28 imaging sessions over a year in the uninjured spinal cord [34], here fibrinolysis induced opacification of the glass window. This prevented us from assessing the dynamics of the inflammatory process at the desired time resolution and forced us to switch to a combination of intravital imaging and post-mortem histology to obtain additional information from our individual subjects at late time points. 

In most studies on biocompatibility, tissue inflammation is evaluated from the global estimation of an undifferentiated pool of macrophages and microglia without separating the peripherally recruited from CNS resident cells [35,36]. Recent transcriptomic studies, however, demonstrated the importance of the crosstalk between peripheral and microglial cells to regulate the post-traumatic inflammatory response to SCI and hence the subsequent functional outcome [16]. Such discrimination is even more crucial when dealing with biomaterials of plasmatic origin, from which the brain is normally shielded. 

In addition to using a triple transgenic mouse model that allowed us to discriminate at least three different subpopulations of CNS recruited inflammatory cells [7,17,22], in the present study, we furthermore took advantage of automated image analysis to discriminate two subpopulations of activated microglial cells based on anatomical features. Ramified morphology is associated with pro-healing activated microglia and round morphology with pro-inflammatory phagocytic microglia, consistent with the need for cell debris clearance prior to the stimulation of axonal regeneration [37,38].

With these tools, we have shown that the initial post-traumatic inflammatory response was governed by two thirds of peripheral myeloid cells and one third of activated microglia. Peripheral inflammation progressively resumed over weeks and let place to microglia only on D90. At an early stage after the lesion, the densities of all immune cell sub-populations were similar in both untreated animals and fibrin treated animals, which suggested that fibrin itself does not impact inflammation until the occurrence of fibrinolysis. Glass window turbidity indicated that it was active during the second week, both in FO animals and, to a lesser extent, in BMF animals.

At a later stage (D90), ramified microglia were found to be the predominant cells in all animals of all conditions compared to the round cells. However, the proportion of these two microglia subtypes differed according to the SCI treatment. In BMFs animals, FDP concentration in the environment was probably not sufficient to enhance chronic microglial activation. In FO animals instead, intensive fibrinolysis was likely responsible for a specific four-fold increase in the phagocytic microglial density compared to untreated animals. Phagocytic microglia were distributed all over the spinal parenchyma rather than confining to the injury site, therefore suggesting the extensive diffusion of FDP from their site of release. Moreover, the presence of phagocytic cells suggested that neurodegeneration was still ongoing at this late post-traumatic stage.

Embedment of the MFs in the fibrin matrix changed the kinetics of fibrinolysis, hence the post-traumatic inflammatory response both at early and late stages. The MFs accelerated the regenerative process by orienting the immune cells towards pro-healing phenotypes, as indicated by a larger proportion of moDCs in BMF animals compared to other conditions. Our results outline the existence of a crucial time window around the end of the first week during which microglia, the main component of chronic inflammation, can interact with infiltrated peripheral immune cells to adopt a pro-regenerative phenotype rather than a sustained pro-inflammatory phenotype with deleterious effects on axons. Several molecular pathways seem to regulate this switch [16,39]. Consequently, on the long term, the MFs reduced the chronic microglial activation observed in FO animals. Our biofunctionalized MFs that successfully bias inflammation might thus be used as local anti-inflammatory treatments for CNS applications, avoiding the systemic side effects of classical pharmacological treatments [40]. 

On the contrary, FDP were here shown as transient M1 immunomodulatory agents, a feature that could not be anticipated from the excellent biocompatibility of polymerized fibrin threads. Our results thus outline the need to assess the biocompatibility of any biomaterial on a long time window after a lesion, as has already been shown for silicone implants by others [41]. Probing the biocompatibility across various life conditions of the recipient subject is required to encounter a large diversity of immunological states and to detect possible side-effects. Rodent models with a short lifespan (about 2 years) are convenient given the homology of their inflammatory response with that observed in human patients [42]. 

### 4.5. Limitation and Prospects

The development of the glial scar is a critical aspect in the context of spinal cord injury, which can affect the implant’s regenerative capacity. Previous studies have indeed shown that astrocytes are the major cellular component of the glial scar that is not just a physical barrier but also plays a crucial role in regulating the inflammatory response and in facilitating axonal regeneration after spinal cord injury [43]. A more in-depth analysis of the glial scar formation using markers such as GFAP or CSPGs [43,44] to characterize microglia-astrocyte interactions and their relationship with the implant should help understand the scaffold’s regenerative potential.

In line with what was described earlier in vivo [7], we showed here that the larger density of individual axons outlined by histological imaging in the presence of MF matched with the enhanced fluorescence ratio observed between the lesioned and uninjured sides of the spinal cord. Having repeatedly observed that fluorescent axonal sprouts quickly disappear or retract over a few hours if severed or non-functional [7,34,45,46,47], we expect this ratio to provide a meaningful estimate of the axonal network state on D90. Additional techniques, such as neural tracing, electron microscopy, and behavioral assays, would however provide more detailed information about axonal morphology, synaptic formation, and functional recovery [48,49]. Noteworthy Basso, Beattie, and Bresnahan (BBB) locomotor rating scales [50] or Rotarod assays are compatible with the presence of spinal glass windows and could also provide a more comprehensive evaluation of the composite implant’s regenerative potential.

## 5. Conclusions

The fibrin and MFs based neuroprosthetic implant appears as a proper therapeutic strategy to manage spinal cord injury. Insertion of bio-functionalized MFs into the lesion has several actions: (1) it boosts the early recruitment of immune cells and promotes the differentiation of moDCs, thereby increasing the amount of anti-inflammatory signals compared to untreated controls; (2) it reduces fibrin sensitivity to plasmin degradation, hence limiting the generation and spread of FDP proinflammatory signals in the tissue, making fibrin a suitable support; and (3) it provides an efficient scaffold to regrowing axons. These should be instrumental for the development of neuroprosthetic implants for therapeutic and regenerative medicine applications [51,52,53] as well as for the development of brain-machine interfaces [54].

## Data Availability

The data presented in this study are available on request from the corresponding authors.
